# Disparities in health-related quality of life between Latino and White men with prostate cancer

**DOI:** 10.1007/s11764-026-01977-8

**Published:** 2026-02-13

**Authors:** Victoria E. Rodriguez, Lorna Kwan, Jiayue Chen, Sarah E. Connor, Mark S. Litwin

**Affiliations:** 1 Department of Obstetrics & Gynecology, David Geffen School of Medicine, University of California, Los Angeles, Box 951740, Los Angeles, CA 90095-1740, USA; 2 Department of Urology, David Geffen School of Medicine, University of California, Los Angeles, Los Angeles, CA, USA; 3 Department of Health Policy and Management, Fielding School of Public Health, University of California, Los Angeles, Los Angeles, CA, USA; 4 School of Nursing, University of California, Los Angeles, Los Angeles, CA, USA

**Keywords:** Prostate cancer, Racial/ethnic disparities, Latino men, Quality of life, Cancer survivorship

## Abstract

**Purpose:**

Among the over 3.5 million prostate cancer survivors in the United States, prostate cancer is frequently diagnosed cancer among Latino men. Latino cancer survivors are more likely to experience poorer quality of life than White survivors. However, limited research has explored quality of life disparities between Latino and White prostate cancer survivors. Hence, we aimed to assess the health-related quality of life between a cohort of Latino and White men with prostate cancer.

**Methods:**

We used survey data from participants in the UCLA Men’s Health Study between 2001 and 2018. Independent t-tests and multivariable linear regressions were used to assess quality of life, both general and prostate-cancer specific between Latino and White men at program enrollment.

**Results:**

Our sample included 291 Latino men and 65 non-Latino White men. In multivariable linear models, general health (*β* = −7.93, *p* < 0.01) and sexual bother (*β* = −14.33, *p* < 0.05) remained significantly worse among Latino men than White men after controlling for age, relationship status, education, income, comorbidities, Gleason grade group, and primary treatment.

**Conclusions:**

Latino men with prostate cancer reported poorer quality of life, particularly in the domains of general health and sexual bother compared to White men.

**Implications for Cancer Survivors:**

Findings highlight the need for survivorship care that addresses Latino prostate survivors’ unique needs including general health and sexual bother.

## Introduction

Of the over 3.5 million prostate cancer survivors in the United States, 33% live at least 10 years and 12% live 20 years or more after their diagnosis [1]. In fact, the 5-year relative survival rate for prostate cancer is 83% for localized or regional stages and 34% for distant stages [2]. Among all stages, prostate cancer has a 10-year survival rate of 98% [2]. These high survival rates are partly due to the usual indolent behavior of prostate cancers, updates in screening guidelines, and advancement in treatment for advanced disease, leading to an increase in overall survival and a growing survivorship population [1].

Among Latino men, prostate cancer has been steadily increasing in the last decade and currently accounts for 25% of all cancers diagnosed [3]. While Latino men have lower incidence of prostate cancer compared to their White counterparts [2], they are more likely to be diagnosed at more advanced stages [3-6]. Further, while the five-year survival for Latino prostate cancer survivors is relatively high, it remains 4% lower than that among Whites [3]. Further, Latino men are often disproportionately impacted by poor social determinants of health such as socioeconomic status, health insurance, and stressors related to discrimination, acculturation, financial burden, and neighborhood context, all of which can negatively impact their overall health and well-being and have cumulative implications for survivorship outcomes including health-related quality of life [7-9].

While the five-year survival is high among prostate cancer survivors, there is substantial evidence of impacts in health-related quality of life for prostate cancer survivors in part due to the long-term effects from cancer treatment [10, 11]. A growing body of research has noted that Latino prostate cancer survivors experience worse quality of life including poorer psychological well-being and suboptimal physical well-being compared to their White counterparts [12-15]. Specifically, research has found that Latino prostate cancer survivors report substantial difficulties with sexual function, urinary function, and bowel function compared to White survivors [16-19]. Literature also shows that Latino prostate cancer survivors who are of lower socioeconomic status experience poorer quality of life [17, 20, 21].

However, survivorship research focused on Latino prostate cancer survivors is lacking in comparison to the burden of disease among this population. Further, exploration into specific domains of health-related quality of life are needed in order to focus future targeted and culturally responsive interventions for Latino prostate cancer survivors. Thus, we aimed to compare the general and prostate-cancer specific health-related quality of life between a cohort of Latino and White men with prostate cancer.

## Methods

### Data source and study population

We used data from the UCLA Men’s Health Study, a prospective cohort of men who enrolled in the Improving Access, Counseling, and Treatment for Californians with Prostate Cancer (IMPACT) program between 2001 and 2018. In 2001, the State of California initiated the IMPACT program (www.california-impact.org) to improve access to prostate cancer care for uninsured or underinsured men with biopsy-proven prostate cancer who live in California and have a household income at or below 200% of the Federal Poverty Level. Men are referred to the IMPACT program through collaborations with community healthcare providers, local health departments, and community-based organizations. Individuals can also access the program through a toll-free telephone line or program website, where they complete eligibility screening prior to IMPACT enrollment. All men enrolled in the IMPACT program are invited to participate in an institutional review board approved companion research study, the UCLA Men’s Health Study (IRB-10–1279). The UCLA Men’s Health Study is a prospective longitudinal cohort of clinical and patient-reported outcomes among low-income men enrolled in the IMPACT program. Participation in the UCLA Men’s Health Study is voluntary and does not impact care provided through the IMPACT program. From IMPACT’s inception in 2001 through December 31, 2018, a total of 2,183 men were enrolled in the clinical program. Of these, 1,323 (60.6% of clinical program participants) consented to participate in the UCLA Men’s Health Study and completed the questionnaire at enrollment.

Participants of the UCLA Men’s Health Study complete a questionnaire that includes demographics, health behaviors, comorbidities, and patient-reported outcomes at study enrollment and every 6 months through 5 years. Questionnaires are completed telephonically with a trained research coordinator and with written surveys in English or Spanish as indicated by participant preference. Participants received a $10 incentive for completing questionnaires up until 2011 when the UCLA Men’s Health Study ended the compensation. Clinical characteristics and treatment information for participants are obtained through medical record abstraction.

### Measures

Outcome variables for this study included general health-related quality of life (HRQOL) and prostate cancer-specific quality of life at study enrollment. General HRQOL was measured using the RAND Medical Outcomes Study Short Form 12-Item Health Survey, version 2 (SF-12). The SF-12 measures general HRQOL and results in two summary scores, physical component summary (PCS) and mental component summary (MCS) [22]. These scores are standardized to the general US population with a mean of 50 and a standard deviation of 10 [22]. Higher scores indicate better HRQOL. Prostate cancer-specific HRQOL was measured using the UCLA Prostate Cancer Index short form (PCI-SF) [23]. The PCI-SF uses 15 items to quantify prostate cancer-specific HRQOL in function and bother for the three domains of urinary, sexual, and bowel [23]. Higher scores on the 0–100 scales indicate better HRQOL [23]. The main independent variable was self-reported race/ethnicity of the participant which was extracted from enrollment questionnaires and dichotomized into Latino and non-Latino White (hereafter referred to as White).

Covariates included demographic and clinical characteristics of participants, extracted from the enrollment questionnaire and medical record abstraction. Demographic covariates included age at enrollment, education level, income, relationship status, and comorbidities. Age at enrollment was treated as a continuous variable. Education level was categorized into three groups: less than high school education, high school graduate, and college graduate (referent group). Total household monthly income was treated as a continuous variable. Relationship status was categorized into three groups: not in a relationship, in a relationship but not living together, and in a relationship and living with spouse/partner (referent group). Comorbidities was categorized into four groups utilizing Charlson Comorbidity Index scores: none (referent group), mild (1–2), moderate (3–4), and severe (≥5) [24]. Clinical characteristics included Gleason score and primary treatment. Gleason score was categorized into grade group (GG): GG1 low-grade cancer (Gleason 3 + 3) (referent group), GG2 medium-grade cancer (Gleason 3 + 4), GG3 medium-grade cancer (Gleason 4 + 3), GG4 high-grade cancer (Gleason sum 8), and GG5 high-grade cancer (Gleason sum 9–10). Primary treatment included four groups: watchful waiting/active surveillance (referent group), hormone therapy, radical retropubic prostatectomy (RRP) including those who also received hormone therapy, and radiation (external beam radiotherapy or brachytherapy) including those who also received hormone therapy.

### Statistical analysis

Descriptive statistics were conducted to examine demographic and clinical characteristics of the sample, and Chi-square and independent t-tests were used to assess differences between Latino and White men. We then conducted independent t-tests to assess HRQOL between Latino and White men. Next, we conducted multivariable linear regressions to assess the HRQOL between Latino and White men after controlling for sociodemographic and clinical covariates. Covariates included in the multivariable models were selected a prior based on theoretical relevance and prior literature, focusing on demographic and clinical characteristics considered important for adjustment. All statistical analyses were performed using Stata 17 (STATA Corp, College Station, TX). The analyses, interpretations, and conclusions in this manuscript are those of the authors, not the State of California.

## Results

### Study population characteristics

A total of 356 men with prostate cancer were included in this study after applying listwise deletion to remove cases with missing data for variables used in the analyses. The majority of the sample (81.7%) was Latino with a mean age of 60.0 (SD 6.7) (Table 1). Participants reported having low socioeconomic status as the majority (55.1%) had less than a high school education and a mean monthly income of $851 (SD = 838). Over half of the sample reported living with their spouse/partner (63.2%) followed by 27.5% not in a relationship, and 9.3% in a relationship but not living together. The majority of the sample (60.7%) reported having no comorbidities. In the sample, 17.1% had GG2 medium-grade cancer, 4.2% had GG3 medium-grade cancer, 7.0% had GG4 high-grade cancer, and 7.3% had GG5 high-grade cancer. The most common treatment was RRP (48.0%), followed by radiation (31.5%), hormone therapy (13.8%), and watchful waiting/active surveillance (6.5%).

Statistically significant differences between Latino and White men were observed in several sociodemographic and clinical characteristics including age, education, and relationship status (Table 1). Latino men were older on average compared to White men (mean (SD): 60.4 (7.0) vs. 58.1 (4.5)). Latino men had less education compared to White men; 65.3% of Latino men had less than a high school education whereas 32.3% and 58.5% of White men were college graduates and high school graduates, respectively. A higher proportion of Latino men (70.1%) were living with a spouse/partner compared to 32.3% of White men. Although not statistically significant, other characteristics such as comorbidities, grade group, and primary treatment also showed notable differences between Latino and White men. White men had a higher proportion of severe comorbidities compared to Latino men (4.6% vs. 1.0%). While Latino men had a higher proportion of GG1 low-grade cancer compared to White men (66.3% vs 55.4%), they also had higher proportions of GG5 high-grade cancer (7.6% vs. 6.2%). Lastly, a larger proportion of Latino men received RRP as their primary treatment compared to White men (49.8% vs. 40.0%), while a higher proportion of White men received radiation as their primary treatment compared to Latino men (36.9% vs. 30.2%).

### Health-related quality of life between Latino and White men

Univariate analyses demonstrated that Latino men with prostate cancer reported worse HRQOL compared to White men (Table 2). Latino men reported significantly worse general health compared to White men (37.7 vs. 44.7; *p* < 0.001). Although differences in PCS and MCS were not statistically significant, Latino men had slightly lower mean PCS (43.9 vs. 46.1; *p* = 0.13) and a slightly higher mean MCS (46.8 vs. 45.2; *p* = 0.31) compared to White men. Significant differences were observed in sexual health with Latino men reporting lower mean sexual function (32.7 vs. 45.1; *p* < 0.01) and lower mean sexual bother (39.6 vs. 55.8; *p* < 0.01) compared to their White counterparts. Latino men also reported significantly lower mean urinary function (72.2 vs. 81.5; *p* < 0.05) compared to White men. Mean urinary bother (62.0 vs. 70.8; *p* = 0.08), mean bowel function (81.4 vs. 84.9; *p* = 0.29), and mean bowel bother (76.0 vs. 80.8; *p* = 0.27) were lower among Latino men compared to White men, but did not differ significantly between groups.

In multivariable models that adjusted for sociodemographic and clinical covariates, Latino men reported general health scores that were 7.9 points lower than those of White men (95% CI: −11.8, −4.1; *p* < 0.01) (Table 3). While Latino men had lower PCS scores of 2.9 (95% CI: −6.3, 0.55; *p* = 0.10) and slightly higher MCS scores of 0.9 (95% CI: −2.7, 4.4; *p* = 0.64) compared to White men, differences were not statistically significant. Within prostate cancer-specific quality of life, Latino men experienced significantly greater sexual bother than White men (*β* = −14.3, 95% CI: −27.6, −1.1; *p* < 0.05). Although Latino men reported lower sexual function (*β* = −7.7; 95% CI: −17.6, 2.3; *p* = 0.13) compared to White men, this difference was not statistically significant. No significant differences were observed between in urinary function (*β* = −2.7; 95% CI: −11.5, 6.0; *p* = 0.54), urinary bother (*β* = −8.8; 95% CI: −20.6, 3.0; *p* = 0.14), bowel function (*β* = −2.0; 95% CI: −9.7, 5.8; *p* = 0.62), or bowel bother (*β* = −6.9; 95% CI: −17.3, 3.5; *p* = 0.19) between Latino and White men.

## Discussion

### Principal findings

Utilizing a prospective cohort of socioeconomically disadvantaged men, this study evaluated differences in HRQOL between Latino and White men with prostate cancer. In unadjusted models, Latino men reported significantly worse general health, sexual function, sexual bother, and urinary function compared to White men. After adjusting for socioeconomic and clinical factors, we found that disparities persisted as Latino men reported significantly worse general and sexual health compared to White men. Our study contributes to a growing body of literature that demonstrates persistent disparities in prostate cancer survivorship outcomes among Latino populations.

We found that even after adjusting for covariates, Latino men reported significantly worse general health compared to White men. Specifically, we found that general health scores reported by Latino men were almost one standard deviation lower (8 points) than those reported by White men. This finding agrees with other studies that have found worse physical and psychological well-being among Latino cancer survivors in the United States [12, 13]. Further, as our sample is representative of low-income men, our findings are congruent with literature that shows worse quality of life among low-income Latino cancer survivors [17, 20]. The disparities we found in general health between Latino and White men may indicate the influence of broader unmeasured social determinants of health and systemic factors such as socioeconomic factors, access to and quality of health-care, immigration status, language, cultural factors, social support, stress, and discrimination [9, 13, 25-28]. Future research might further examine the factors that may be associated with worse general health outcomes of Latino men with prostate cancer in order to develop targeted interventions to mitigate these quality of life disparities. Interventional efforts may include improving access to survivorship care and addressing systemic barriers that contribute to poorer outcomes.

We also found that after adjusting for covariates, Latino men reported more sexual bother compared to White men. We found that mean sexual bother was 14 points lower for Latino than White men. While limited in the literature, previous studies focused on prostate cancer-specific quality of life among Latino men have had differing findings. These inconsistencies may reflect differences in study populations (e.g., disease stage, treatment type, timing of assessment), as well as cultural or linguistic factors that influence how men interpret and report sexual bother and function. For instance, one study focused on Latino men with localized prostate cancer reported significant challenges in sexual functioning [16]. Another study among men treated with radical prostatectomy found that Latino men were more likely to report sexual function compared to their White counterparts [29]. Similarly, a prospective population-based study focused on localized prostate cancer found that at baseline, Latino men reported lower sexual function scores compared to White men [19]. However, results from research among men on active surveillance found that Latino men reported less sexual bother compared to White men [17]. Although contradictory findings have been reported, the limited research on this topic and the results of this study highlight the need for additional research to clarify whether a true disparity exists and to better understand the cultural and contextual factors that may shape sexual health reporting among Latino men with prostate cancer. Although unmeasured in our study, past qualitative literature have indicated that stigma and cultural contexts can potentially impact the ways in which Latino men conceptualize prostate cancer and in turn may impact their communication and discussions about sexual health issues [30-33]. This underscores the importance of future studies that more directly examine culturally informed communication patterns and contextual factors related to sexual health among Latino prostate cancer survivors.

Our study has several limitations. First, due to limitations of the data we were unable to disaggregate Latino subgroups to assess whether HRQOL and disparities in HRQOL differed by Latino subgroup. Future research should assess subgroup differences in HRQOL among Latino men in order to identify subgroups most impacted. Second, although the SF-12 is a widely accepted measure of HRQOL, its brevity limits its comprehensiveness as it generates only summary physical and mental component scores [22]. Therefore, it does not capture detailed HRQOL domain-level information such as more nuanced aspects of social functioning, pain, and role limitations. Third, although the HRQOL instruments we used have been rigorously tested for linguistic and cultural appropriateness [34, 35], there may still be a cultural bias in the way that Latino men interpreted and responded to questions which may have led to an underestimation of the study results. Fourth, our sample was drawn from men enrolled in the IMPACT program, which included participants at different stages of their cancer trajectory (i.e., before or after treatment). This variation could have introduced bias or misclassification, as clinical characteristics for some participants had to be retrospectively ascertained. Fifth, the use of self-reported information in questionnaires introduces the potential for recall or reporting bias. Participants may have had difficulty accurately recalling past experiences or may have under- or over-reported certain information due to stigma, social desirability, or personal interpretation of the questions. These sources of measurement error could lead to misclassification and potentially attenuate the observed associations. Sixth, several potentially relevant covariates such as language, employment, and cancer stage had a substantial amount of missing or unreported data and therefore could not be included in the adjusted analyses. This limitation may reduce our ability to fully account for sociodemographic and clinical differences that could influence HRQOL outcomes. Future studies should aim to include and adjust for more comprehensive data on social, structural, and clinical factors that are needed to better understand differences in HRQOL between Latino and White men with prostate cancer. Lastly, the IMPACT program specifically targets low-income, uninsured or underinsured men from California, therefore our study findings may not be representative or generalizable to other populations or geographic regions.

Taken together, our findings underscore the importance of culturally appropriate survivorship care that is responsive to the needs of Latino men with prostate cancer. Although additional research is needed to clarify the nature and drivers of disparities in sexual health for Latino men, future interventions may ultimately benefit from incorporating culturally grounded approaches that enhance access to supportive care and address both general health and sexual health concerns. In addition, longitudinal studies are needed to understand how HRQOL changes over time and to identify key timepoints at which targeted support may help mitigate emerging disparities in survivorship.

## Figures and Tables

**Table 1:** Participant characteristics (*N* = 356)

	Total (*N* = 356)	Latino men (*n* = 291)	White men (*n* = 65)	*p*-value
Age, mean (SD)	60.0 (6.7)	60.4 (7.0)	58.1 (4.5)	**0.01**
Education level, % (*n*)				**0.00**
Less than high school education	55.1 (196)	65.3 (190)	9.2 (6)	
High school graduate	33.2 (118)	27.5 (80)	58.5 (38)	
College graduate	11.8 (42)	7.2 (21)	32.3 (21)	
Total household monthly income, mean (SD)	851.0 (838.1)	857.4 (846.0)	822.6 (807.6)	0.76
Relationship status, % (*n*)				**0.00**
Not in a relationship	27.5 (98)	20.6 (60)	58.5 (38)	
In a relationship, not living together	9.3 (33)	9.3 (27)	9.2 (6)	
Living with spouse/partner	63.2 (225)	70.1 (204)	32.3 (21)	
Comorbidities, % (*n*)				0.23
None	60.7 (216)	61.5 (179)	56.9 (37)	
Mild	34.3 (122)	34.0 (99)	35.4 (23)	
Moderate	3.4 (12)	3.4 (10)	3.1 (2)	
Severe	1.7 (6)	1.0 (3)	4.6 (3)	
Grade group (GG), % (*n*)				0.11
GG1 Low-grade	64.3 (229)	66.3 (193)	55.4 (36)	
GG2 Medium-grade	17.1 (61)	16.2 (47)	21.5 (14)	
GG3 Medium-grade	4.2 (15)	4.5 (13)	3.1 (2)	
GG4 High-grade	7.0 (25)	5.5 (16)	13.9 (9)	
GG5 High-grade	7.3 (26)	7.6 (22)	6.2 (4)	
Primary treatment, % (*n*)				0.55
Watchful waiting/active surveillance	6.5 (23)	6.2 (18)	7.7 (5)	
Hormone therapy	13.8 (50)	13.8 (40)	15.4 (10)	
Radical retropubic prostatectomy (RRP)	48.0 (171)	49.8 (145)	40.0 (26)	
Radiation	31.5 (112)	30.2 (88)	36.9 (24)	

**Table 2:** Univariate analysis of health-related quality of life between Latino and White men with prostate cancer (*N* = 356)

	Latino men (*n* = 291)		White men (*n* = 65)		*p*-value
	Mean	SD	Mean	SD	
SF-12					
General health	**37.7**	11.8	**44.7**	12.2	**0.00**
Physical composite score	43.9	10.0	46.1	12.6	0.13
Mental composite score	46.8	11.5	45.2	8.9	0.31
UCLA PCI					
Sexual function	**32.7**	30.9	**45.1**	37.4	**0.00**
Sexual bother	**39.6**	41.0	**55.8**	40.9	**0.00**
Urinary function	**72.2**	29.7	**81.5**	24.0	**0.02**
Urinary bother	62.0	36.9	70.8	35.5	0.08
Bowel function	81.4	24.9	84.9	19.1	0.29
Bowel bother	76.0	32.6	80.8	27.9	0.27

**Table 3:** Multivariable linear model of health-related quality of life among men with prostate cancer (*N* = 356)

	*β*	95% CI	*p*-value
SF-12 General health			
White men	Referent	-	-
Latino men	**−7.9**	**(−11.8, −4.1)**	**0.00**
SF-12 Physical component summary			
White men	Referent	-	-
Latino men	−2.9	(−6.3, 0.6)	0.10
SF-12 Mental component summary			
White men	Referent	-	-
Latino Men	0.9	(−2.7, 4.4)	0.64
UCLA PCI sexual function			
White men	Referent	-	
Latino men	−7.7	(−17.6, 2.3)	0.13
UCLA PCI sexual bother			
White men	Referent	-	
Latino men	**−14.3**	**(−27.6, −1.1)**	**0.03**
UCLA PCI urinary function			
White men	Referent	-	
Latino men	−2.7	(−11.5, 6.0)	0.54
UCLA PCI urinary bother			
White men	Referent	-	
Latino men	−8.8	(−20.6, 3.0)	0.14
UCLA PCI bowel function			
White men	Referent	-	
Latino men	−2.0	(−9.7, 5.8)	0.62
UCLA PCI bowel bother			
White Men	Referent	-	
Latino Men	−6.9	(−17.3, 3.5)	0.19

All models controlled for age, education level, income, relationship status, comorbidities, grade group, and primary treatment

## Data Availability

The datasets analyzed in the current study are not publicly available.
